# Movements of a juvenile Crowned Eagle (*Harpyhaliaetus coronatus*) tracked by satellite telemetry in central Argentina

**DOI:** 10.1186/2241-5793-21-12

**Published:** 2014-07-01

**Authors:** Vicente Urios, Maria Pilar Donat-Torres, Mark Bechard, Miguel Ferrer

**Affiliations:** Estación Biológica Terra Natura, Universidad de Alicante, Alicante, E-03080 Spain; Instituto de Investigación para la Gestión Integrada de Zonas Costeras IGIC, Universidad Politecnica de Valencia, Gandia, E-46730 Spain; Department of Biology, Boise State University, Boise, ID 83725-1515 USA; Departamento de Etología y Conservación de la Biodiversidad, Estación Biológica de Doñana, Consejo Superior de Investigaciones Científicas CSIC, Sevilla, E-41092 Spain

**Keywords:** Conservation, Dispersal, Raptors, GIS

## Abstract

**Background:**

A juvenile Crowned Eagle was tagged at its nest with a satellite transmitter. The Crowned Eagle (*Harpyhaliaetus coronatus*) is one of the most unknown raptor species from the American continent. Their current distribution ranges from central Brazil to central Argentina, with a total population of 350–1500 individuals across this large area, being thus largely fragmented.

**Results:**

During the three years of tracking the bird concentrated its movements in a range spanning for 12845 km^2^, but concentrating mainly in four smaller areas accounting for 3073 km^2^. The locations were recorded mainly over shrubland habitats (86.5%), whereas other habitats used were different types of mosaics that included cropland and natural vegetation (forest, shrubland or grassland) close to wetlands.

**Conclusions:**

The home-range estimated for this individual during the whole period was 12845 km^2^ (according to 95% fixed kernel). However, the bird concentrated most of its movements in smaller areas (as defined above), that accounted for a total of 3073 km^2^ (50% fixed kernel). During these three years, most of the locations of the juvenile solitary Crowned Eagle were recorded over shrubland habitats (86.5% of the locations). Understanding in a more detailed way the juvenile ranging behaviour and habitat preferences would be of great importance for the conservation of the Crowned Eagle.

## Background

Juvenile dispersal represents the movements undertaken by juvenile animals to find a breeding site, once they become independent from their parents [1]. In large raptors, this period is one of the most unknown stages in their life (e.g. [2]), mainly due to the difficulty in tracking the movements of dispersing birds [3]. This is especially difficult in non-philopatric species, as absence of proper monitoring schemes prevents to get proper data on this, particularly when observational data is used [4]. Nevertheless, the fate of juvenile birds during this period may have important consequences for population dynamics and thus, it is of particular importance from a conservation point of view [5–8]. The Crowned Eagle (*Harpyhaliaetus coronatus*) is one of the most unknown raptor species from the American continent [9, 10]. Their current distribution ranges from central Brazil to central Argentina, with a total population of 350–1500 individuals across this large area, being thus largely fragmented [10]. Main threats for the species are habitat destruction (e.g. intensive cattle ranching, forest clearance or invasive grasslands) and direct persecution, especially hunting [10–13]. Although population trends are difficult to detect in such low density populations, and given the severity of threats, it seems likely that a significant population decrease is occurring across the whole species’ distribution range. For this reason, the Crowned Eagle is currently globally listed as Endangered Species [10]. In Argentina, most of the records of the species belong to the "Monte" ecoregion [12]. The majority of the observations corresponded to solitary individuals and pairs but also groups of up to three individuals composed of two adults and one year-old juvenile have been observed [14, 15]. Bellocq *et al.* [16] indicated that habitat preferences for the species are mainly determined by the availability of tall isolated trees, which are used for nesting and also as roosts. However, movements of individuals during the juvenile dispersal period of the species are largely unknown.

This species share the same habitat with other resident raptors, especially the Crested Caracara (*Plyborus plancus*), but also the Chimango Caracara (*Milvago chimango*), the Peregrine Falcon (*Falco peregrinus*), the Spot-winged falconet (*Spiziapteryx circumcinctus*) and the Aplomado Falcon (*Falco femoralis*). Among scavengers using this habitat we can find the Turkey Vulture (*Cathartes aura*) and the American Black Vulture (*Coragyps atratus*). Within the family *Accipitridae*, the present species are the Cinereous Harrier (*Circus cinereus*), the White Tailed Hawk (*Buteo albicaudatus*), the Rufous Tailed Hawk (*Buteo ventralis*) and the Red Backed Hawk (*Buteo polysoma*). These species are joined by the Swainson's Hawk (*Buteo swainsoni*) during the austral summer, while during the austral winter also the White Tailed Kite (*Elanus leucurus*), the Long-Winged Harrier (*Circus buffoni*) and the Savanna Hawk (*Buteogallus meridionalis*) do occur [9]; therefore the only "true eagle" species included in this raptor community is the Crowned Eagle.

In recent years, satellite telemetry has provided new important insights into the juvenile dispersal ecology and habitat use of poorly-known large raptors, even when few individuals are tracked (e.g. [17–20]). Here, we describe the movements undertaken by a juvenile Crowned Eagle tracked by satellite telemetry for three years since it was tagged at its nest. We also assess the habitats used by this individual during the study period. To our knowledge, these are the first detailed data concerning the juvenile dispersal period of this species, which may be important from a conservation point of view.

## Results and discussion

**Figure 1:**
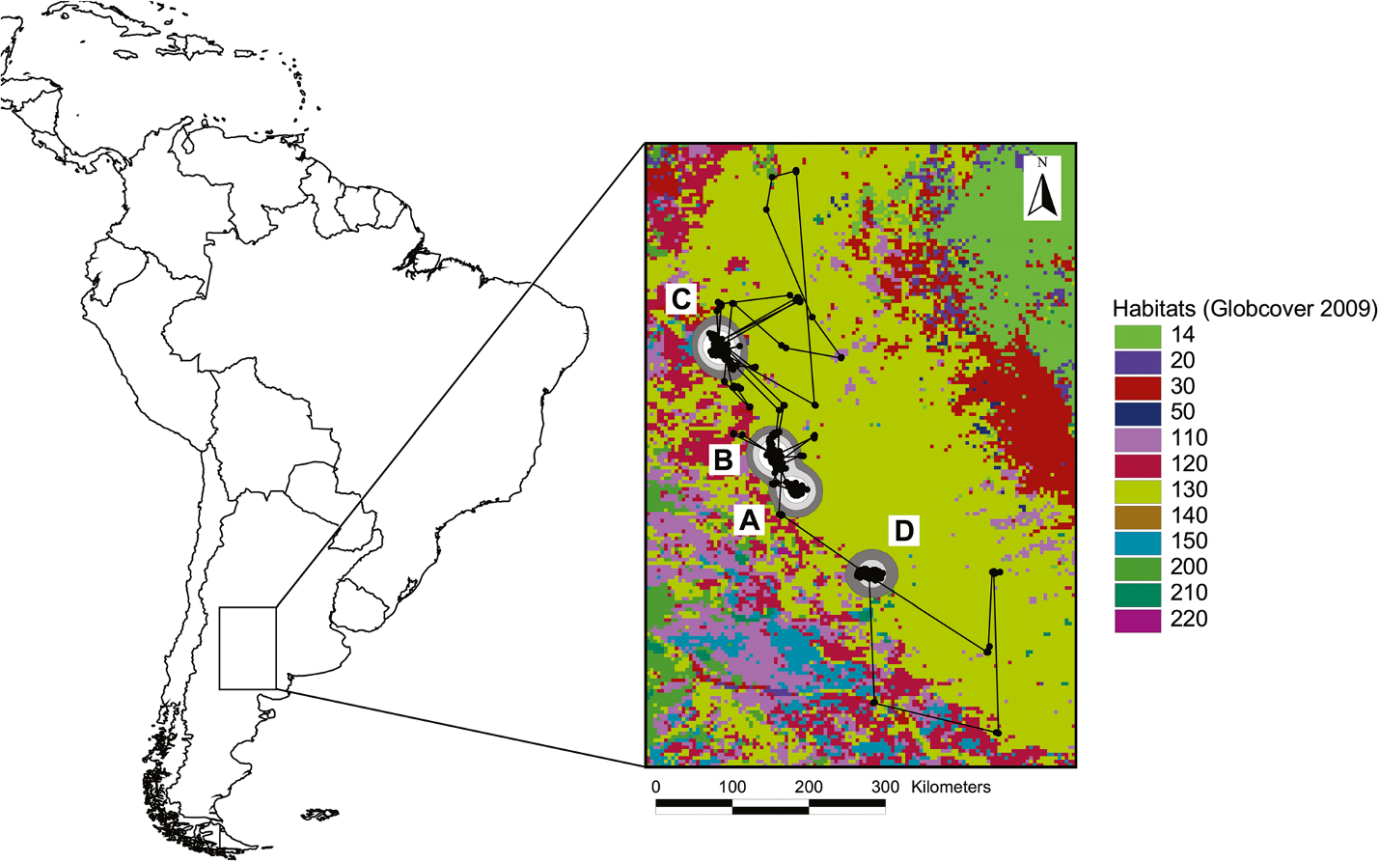
**Movements of a satellite tracked juvenile Crowned Eagle in central Argentina.** Locations are indicated by black dots and polygons represent fixed kernels (95%: dark grey, 75%: light grey and 50%: white). The eagle was tagged at the nest in zone A, and subsequently moved to zones B, C and D (see text for details). Habitat categories according to GlobCover global land cover map V.2.3 are the following. 14: Rainfed croplands; 20: Mosaic cropland (50-70%)/vegetation (grassland/shrubland/forest) (20-50%); 30: Mosaic vegetation (grassland/shrubland/forest) (50-70%)/cropland (20-50%); 110: Mosaic forest or shrubland (50-70%)/grassland (20-50%); 120: Mosaic grassland (50-70%)/forest or shrubland (20-50%); 130: Closed to open (>15%) (broadleaved or needleleaved, evergreen or deciduous) shrubland (<5 m); 150: Sparse (<15%) vegetation; 200: Bare areas; 210: Water bodies.

During the study period, a total of 1315 high-quality locations (as defined in Methods) from the tracked eagle were received (i.e., retained for the analyses). Movements of the eagle during the study period are illustrated in Figure 1. Initially, the bird moved short distances, staying 259 days in the nesting area (until 5th October 2007) and moving in a radius of *ca.* 7 km (zone A in Figure 1). Then, it moved to another area located at *ca.* 50 km to the northwest of the natal area, where it settled on 12th October 2007 and spent 276 days (until 15th July 2008) and mainly moved in a radius of 15 km (zone B in Figure 1). From this area, the juvenile eagle still continued moving to the northwest, and settled in a new area located 150 km away from the previous zone, and it spent there more than a year (383 days, from 22nd July 2008 to 9th August 2009; zone C in Figure 1). While being in this area, the bird performed the largest exploratory movements of the whole study period, with a maximum distance to the centre of this area of 246 km, but always returning to that area. Finally, the eagle moved to the southeast, spent 14 days on the zone B (between 12th and 26th August 2009), and then moved to a new area located *ca.* 140 km to the southeast of the natal area (zone D in Figure 1), where it arrived on 2nd September 2009 and remained there at least until the PTT stopped transmitting on 19th January 2010 (139 days), moving mostly in a radius of *ca.* 10 km.

Table 1 shows the percentage of locations (N = 1315) recorded within different habitat types used by the tracked juvenile Crowned Eagle during three years and percentages of random points. The comparison with available habitat was significant (*χ*^2^ = 49.1, g.l. = 8, *p* < 0.05), but this was mainly due to the absence of observed locations from not abundant (≤1%) habitats (Table 1). A further inspection of locations on Google Earth revealed that these activity areas (according to 50% and 75% fixed kernels) were mainly located close to wetlands and small lakes along the Atuel and Salado rivers. Concerning seasonal differences, they were significant (*χ*^2^ = 67.6, g.l. = 12, *p* < 0.05), but mainly because of underrepresented habitats, since shrubland habitats were by far always the most used ones (Table 2).

**Table 1:** **Percentage of locations and percentages of random points**

Habitat	% locations	% random points
Mosaic vegetation (grassland/shrubland/forest) (50-70%)/cropland (20-50%)	1.2	1.1
Mosaic forest or shrubland (50-70%)/grassland (20-50%)	3.9	3.9
Mosaic grassland (50-70%)/forest or shrubland (20-50%)	6.7	5.9
Closed to open (>15%) (broadleaved or needleleaved, evergreen or deciduous) shrubland (<5 m)	86.5	86.8
Bare areas	1.7	0.4
Sparse (<15%) vegetation	0	1.0
Mosaic cropland (50-70%)/vegetation (grassland/shrubland/forest) (20-50%)	0	0.3
Water bodies	0	0.5
Rainfed croplands	0	0.1

Percentage of locations (N = 1315) recorded within different habitat types used by the tracked juvenile Crowned Eagle during three years and percentages of random points.

**Table 2:** **Locations within different habitat types**

Habitat	Summer	Autumn	Winter	Spring
Mosaic vegetation (grassland/shrubland/forest) (50-70%)/cropland (20-50%)	4	0	12	0
Mosaic forest or shrubland (50-70%)/grassland (20-50%)	11	11	4	25
Mosaic grassland (50-70%)/forest or shrubland (20-50%)	21	30	13	24
Closed to open (>15%) (broadleaved or needleleaved, evergreen or deciduous) shrubland (<5 m)	299	281	288	269
Bare areas	4	4	1	14

Locations recorded within different habitat types used by the tracked juvenile Crowned Eagle according to the season in the austral hemisphere.

Despite our results derived from only one tracked individual, it has been followed during its movements for three years and, to the best of our knowledge, it has been the first time that a Crowned Eagle has been tracked by means of satellite telemetry. Moreover this species shows a very low density population where only few hundred pairs are still active [10]. Therefore we believe that our results provide valuable information to promote further studies and conservation actions on this species.

In large raptors with delayed maturity and large home-ranges, the process of juvenile dispersal until establishing in a breeding territory could take several years (e.g. [2, 17, 20]). Threats faced by juvenile birds during this period may have important consequences for population dynamics and hence, any action aimed at getting information on this may be important from a conservation point of view. Here, in spite of the restrictions imposed by the small sample size, our results are the first quantitative description of the ranging behaviour of a juvenile Crowned Eagle during the juvenile dispersal stage. Despite that we do not know whether the tracked Crowned Eagle finally bred, the ranging behaviour of the tracked juvenile appears to be similar to those of other large eagles of temperate regions, such as Bonelli’s (*Aquila fasciata*) and Spanish Imperial eagles (*Aquila adalberti*). Juveniles of these species typically follow a "far-sighted", oriented strategy, restricting their movements to a few temporary settlement areas that are regularly used for hunting and roosting [19, 21–24]. In contrast, other large eagles, such as golden Eagle (*Aquila chrysaetos*), follow a "blind", un-oriented strategy, continuously increasing the size of their dispersal area, exploring new territories throughout their juvenile dispersal stage [25, 26]. This un-oriented strategy is the most demanding in terms of energy requirements [27], and thus can only be employed by animals that can alternatively exploit food resources abundant enough to fulfil individuals’ energy requirements. Alternatively, when energy sources are less abundant, an oriented strategy could be more effective in energetic terms [27, 28]. Given that oriented movements are an efficient strategy for locating suitable foraging habitats [28], the juvenile Crowned Eagle could be restricting its movements to a network of sites that ensure high chances of successful hunting [18, 29]. The juvenile eagle tracked here used mostly shrublands during the study period, as well as flooded areas along rivers. This is in agreement with a previous study, where the species was reported to actively use woodlands or isolated trees to rest, which were interspersed in a shrubland matrix [16]. These small forest remnants within larger shrublands are generally used for nesting and therefore, this is also an important habitat for the species from a conservation point of view [16, 30]. In fact, the lost of trees for nesting due to the incidence of fires and also due to changes in land use could be affecting the reproduction of this species [12]. On the other hand, the use of wetlands and small lakes is probably related to a higher productivity in those areas compared to that in the surrounding habitats, as it happens with other large raptors in temperate semi-arid areas [31, 32]. Hence, we show here that other habitats than woodlands used during the nesting period may be important for the species throughout their life-cycle and hence, they need to be considered into conservation schemes for this species. These more-open shrubland habitats could be used by juvenile eagles (as shown here), but may be also important for adult birds during the nesting period [16].

The results of satellite tracking studies are usually limited because of the small sample size associated with the methodology. However, obtaining habitat related information about birds’ movements is of great importance from a conservation point of view [33–36], even when few birds are tracked (e.g. [19, 20, 37]). In the case of the Crowned Eagle, further studies involving a higher number of individuals tracked could provide more insights into the juvenile dispersal stage of the species, for example to properly determine the onset of dispersal (e.g. [26, 38]), as well as to provide a more detailed understanding of the habitat preferences and requirements of this endangered species. Other interesting questions to be analyzed with conservation concerns could be to evaluate the existence of sexual differences in relation to ranging behaviour and to track individuals of different populations with the aim of estimating the levels of connectivity between subpopulations and thus, to describe the metapopulation structure and dynamics of the highly fragmented population of this species.

## Conclusions

The home-range estimated for this individual during the whole period was 12845 km^2^ (according to 95% fixed kernel). However, the bird concentrated most of its movements in smaller areas (as defined in Methods), that accounted for a total of 3073 km^2^ (50% fixed kernel). During these three years, most of the locations of the juvenile solitary Crowned Eagle were recorded over shrubland habitats (86.5% of the locations), whereas other habitats used were different types of mosaics that included cropland and natural vegetation (forest, shrubland or grassland).

A proper understanding of the juvenile ranging behaviour and habitat preferences would be of great importance for the conservation of the Crowned Eagle.

## Methods

### Study area

The study area covers part of the departments of Chical-Co, and Limay Mahuida Chalileo in the province of La Pampa, entering from the north in the province of Mendoza. It belongs to the Monte biogeographic province [39] and geomorphological subregion called "the river floodplains Atuel". The river empties into the Andean Atuel Desagüadero-Salado river forming a floodplain with dunes, plains and sand ridges interspersed with residual plateaus. In general, soils are Entisols. The climate is semi-arid, with average annual temperatures of 15°C and rainfall around 300 mm. It is sparsely populated, agricultural and livestock activities being bovine and caprine cattles. The vegetation is characterized by the predominance of open shrublands [40]. The open shrub *Larrea divaricata* have stood where sand ridges, and other species can codominar: *Chuquiraga erinacea*, *L. divaricata*; *Prosopis flexuosa*. There may be presence of trees and mixed forests formation (*Prosopis caldenia*) broadleaved or needleleaved in the cooler areas. The shrubland of *Atriplex lampa* occupies areas peripheral to the salt flats where the salt content is low. The shrubland of *Cyclolepis genistoides* extends on the periphery of saline areas, forming a ring or belt or semihalófila halophytic vegetation. The halophyte *Atriplex undulata* scrub supports immersion periods of heavy rainfall. Helophytic vegetation (*Typha subulata*, *Phragmites australis*) is installed in permanently flooded areas. Also common psammophile grasslands (*Stipa tenui*s, *Piptochaetium napostaense*) transformed by grazing that vary based on usage floristically agriculture.

### Field procedures and data analysis

The juvenile Crowned Eagle was trapped on 19th January 2007 at its nest in central Argentina (-36.708°, -66.968°; province of La Pampa), when it was 40–50 days-old. It was tagged with a satellite transmitter (PTT) manufactured by North Star (North Start Science and Technology, LLC, King George, VA, USA), which was affixed to their backs using a Teflon harness (e.g. [41]). Duty cycle of the PTT was set to 12 h ON/48 h OFF (approximately to collect data for one day every three days). Bird was tracked for three years (until 19th January 2010), when the PTT stopped working for unknown reasons. The juvenile eagle was tracked using the Argos satellite tracking system [42], which estimate locations and calculates an error distribution for each location (i.e. measure of likely accuracy, known as location class -LC-). Nominal high-quality locations (LC 3, 2 and 1) are usually used for analyses that require high degree of accuracy such as those related to habitat use [43], although locations of nominally lower quality (0, A and B) can be used to analyze large-scale movements (such as migrations; e.g. [43–45]). Here we used only locations belonging to LCs 3 to 1 to describe movements in this period as well as for further analyses. Also, when more than one location was received within an hour, we only retained that of higher quality and the others were excluded from the dataset to avoid spatial and temporal autocorrelation (e.g. [41]). We estimated the home-range of the juvenile eagle during the study period using a fixed kernel approach considering all locations received in that period [46]. The 95%, 75% and 50% fixed kernels were calculated using the Animal Movement extension for ArcView 3.2 [47], and using the LSCV procedure [48] to calculate the smoothing parameter (H). The actual size of each kernel polygon was calculated after projecting the shapes into an Equal-Area Cylindrical projection of the globe using the Projector! extension for ArcView 3.2. Finally, we assessed habitats used by the juvenile eagle using the GlobCover global land cover map V.2.3 [49], available in a raster format of 300 m resolution and obtained from satellite images recorded during 2009. We rescaled this map to a 2.5 arc-min pixel size (i.e., 4.5 × 4.5 km) by retaining the main cover (mode of the pixels within) as the descriptor of the new pixels, to account for the accuracy of Argos-based satellite locations (e.g. [43, 50]). We assigned a habitat type to every location recorded during the study period and then calculated the proportions of fixes in every habitat type used. We also compared the observed proportions with expected ones, obtaining habitat availability through 3000 random points within the Minimum Convex Polygon of the observed data [43]. Finally, we checked for seasonal differences comparing the proportions of different habitats across the four seasons. These comparisons were made using Chi-square contingency tables.

## Abbreviations

PTT: Platform Terminal Transmitter
LLC: Limited Liability Company.
